# Planktonic prey size selection reveals an emergent keystone predator effect and niche partitioning

**DOI:** 10.1371/journal.pone.0280884

**Published:** 2023-02-13

**Authors:** Darcy A. A. Taniguchi, Michael J. Follows, Susanne Menden-Deuer

**Affiliations:** 1 Massachusetts Institute of Technology, Cambridge, MA, United States of America; 2 California State University San Marcos, San Marcos, CA, United States of America; 3 University of Rhode Island, Narragansett, RI, United States of America; National Taiwan University, TAIWAN

## Abstract

Marine herbivorous protists are often the dominant grazers of primary production. We developed a size-based model with flexible size-based grazing to encapsulate taxonomic and behavioral diversity. We examined individual and combined grazing impacts by three consumer sizes that span the size range of protistan grazers– 5, 50, and 200 μm—on a size-structured phytoplankton community. Prey size choice and dietary niche width varied with consumer size and with co-existence of other consumers. When all consumer sizes were present, distinct dietary niches emerged, with a range of consumer-prey size ratios spanning from 25:1 to 0.4:1, encompassing the canonical 10:1 often assumed. Grazing on all phytoplankton size classes maximized the phytoplankton size diversity through the keystone predator effect, resulting in a phytoplankton spectral slope of approximately -4, agreeing with field data. This mechanistic model suggests the observed size structure of phytoplankton communities is at least in part the result of selective consumer feeding.

## Introduction

Herbivorous protists are the dominant grazers of phytoplankton in much of the world’s oceans [1, 2]. Through grazing, protists have the potential to impact planktonic community size structure and composition, nutrient regeneration, carbon export, and food webs [3–6]. The diversity of species, feeding interactions, and prey preferences have made mathematical abstraction of protist grazing for ecosystem models difficult [7, 8], which leaves the single largest loss factor of marine primary production poorly constrained in ecosystem models.

Model representations of protist grazing often rely on assumptions that obscure consumer diversity and behavior. For example, protists may be considered indistinct from mesozooplankton (e.g., [9]), constrained to graze specific prey types (e.g., [10]), or prescribed to graze prey approximately 1/10 the consumer’s size [11–13]. While these assumption are valid for many predator-prey relationships in the plankton, protists have diverse feeding types with broad prey size spectra [14–17], sometimes including prey of equal or larger size than the consumer [18–20]. For example, literature reviews of protistan consumers indicate that predator-prey size ratios can be both above and below 1 [16, 17]. Furthermore, grazing rates often only provide implicit information about prey [13, 21, 22]. Constraining consumer feeding behaviors and prey size ratios may obscure the mechanistic underpinnings of the effects consumers have on phytoplankton communities.

Progress has been made to decipher the individual steps involved in planktonic grazing, and idealized models that incorporate the details of the grazing process help determine the underlying mechanisms that impact the planktonic community. For example, protistan grazing has been broken down into the distinct but linked steps of searching, contact, capture, processing, ingestion, and digestion [23]. Weisse et al. [15] suggested that encounter rates and processing time drive the bulk of the numerical and functional responses of protists. Mitra et al. [24] found that maximum ingestion and assimilation influenced temporal changes in predator and prey biomass. Banas [25] found that the degree of prey selectivity in single and multicellular grazers affected the variability in phytoplankton biomass, which additionally varied with the timescale. Incorporating details of the feeding process thus has a demonstrable effect on phytoplankton abundance and composition.

Building upon these efforts, we have developed a mechanistic model specifically focused on single-celled eukaryotic grazers, a term we have used interchangeably with consumers and herbivorous protists. This model represents planktonic diversity via different size classes and includes formulations based on first principles of consumers encountering and processing prey. Compared to simpler, more prescriptive models, this flexible, empirically driven framework allows for the diversity of protistan behavior to emerge and the ability to examine the mechanistic underpinnings of the emergent results.

Here we have modeled herbivorous protist feeding behavior but recognize there are other forms of resource acquisition besides herbivory [26, 27]. In this mechanistic model, we used parameterizations that reflect empirical data specific to protistan behaviors (e.g., broad prey size spectra, e.g. [15–17]). As more parameterizations emerge, different functional forms and values can be incorporated to reflect this growing knowledge.

Within this framework, we examined the 1) prey preferences for different size consumers and 2) how prey choice influenced phytoplankton abundances and biomass spectra. We show how this model reflects properties of natural communities, including decreasing size spectra [28–30], the keystone predator effect [31, 32], and dietary niche partitioning [33, 34]. These insights highlight the top-down control herbivorous protists can have on plankton community structure.

## Materials and methods

### Nutrient-phytoplankton-zooplankton model framework

We used a nutrient-phytoplankton-zooplankton (NPZ) model framework [35], modified to include size (e.g., [22, 36]), represented as the cell diameter *s*:

$$\begin{array}{c} \frac{dP(s_{i})}{dt}=P\left(s_{i}\right)[\mu \left(s_{i}\right)\frac{N}{N+k_{s}\left(s_{i}\right)}-g\left(s_{j},s_{i}\right)\frac{Z(s_{j})}{P\left(s_{i}\right)+k_{z}\left(\beta ,Q_{p,i},h,c\right)}-m_{p}], \end{array}$$ (1)

$$\begin{array}{c} \frac{dZ(s_{j})}{dt}=Z\left(s_{j}\right)[\gamma g\left(s_{j},s_{i}\right)\frac{P(s_{i})}{P\left(s_{i}\right)+k_{z}\left(\beta ,Q_{p,i},h,c\right)}-R\left(s_{j},v,Q_{z,j}\right)-m_{z}\left(s_{j}\right)], \end{array}$$ (2)

$$\begin{array}{c} N_{T}=N+\sum_{i}^{n}P(s_{i})+\sum_{j}^{m}Z(s_{j}), \end{array}$$ (3)

Eqs (1), (2) and (3) describe the change with time *t* of the three state variables phytoplankton biomass *P*, consumers biomass *Z*, and dissolved nutrient concentration *N* (Table 1). Eq (1) describes the change in *P* for different size classes via growth, grazing, and general mortality (symbols and dimensions in Table 2). Consumer biomass (Eq 2) varies with grazing intake, respiration losses, and general loss (Tables 2 and 3). In this closed system, total nutrients *N*_*T*_ (Eq 3) is the sum of dissolved nutrients *N* and nutrients in all phytoplankton and consumer size classes. Given our explicit inclusion of respiration, the model is parameterized with carbon. However, we note that, throughout the entire model, we assume a Redfield ratio of 106 C: 16 N: 1 P. Therefore, the choice of carbon is made for consistency across all model parameters, and output and can be converted to nitrogen or phosphorous using the appropriate conversion from the Redfield ratio. We elaborate on this point in the section below.

**Table 1:** Model state variables. Model variables for the phytoplankton biomass, grazer biomass, nutrients, time, and size.

Variable	Units	Description
*t*	s	time
*P*	mM C	phytoplankton biomass
*s*_*i*_	μm	phytoplankton diameter for size class i
*Z*	mM C	herbivorous protist biomass
*s*_*j*_	μm	herbivorous protist diameter for size class j
*i*	No units	index for phytoplankton size class
*j*	No units	index for herbivorous protist size class
*N*	mM C	dissolved nutrients
*N*_*T*_	mM C	total nutrients

**Table 2:** Size-dependent variables. The coefficient and exponent values typically correspond to *a* and *b*, respectively, in the allometric relationship *r* = *as*^*b*^ for *s* the diameter of the cell in μm and *r* the rate or property. Exceptions include *g*, which takes the form in Eq (4), *h*, which takes the form in Eq (5), and *c*, which takes the form in Eq (7). NA means not applicable.

Variable	Units	Coefficient	Exponent	Description
*μ*	day^-1^	1.36	-0.16	maximum phytoplankton growth rate
*k*_*s*_	mM C	2.19 x 10^-3^	0.48	phytoplankton nutrient half saturation constant
*g*	day^-1^	264.70	-1.83	herbivorous protist max grazing rate
*Q*_*p*,*i*_	mmol C (phyto cell)^-1^	9.8 x 10^-12^	2.82	carbon per phytoplankton cell i
*Q*_*z*,*j*_	mmol C (grazer cell)^-1^	9.8 x 10^-12^	2.82	carbon per herbivorous protist cell j
*h*	day	4.4 x 10^-3^	-1.01	handling time
*m*_*p*_	day^-1^	5.0 x 10^-3^	0	phytoplankton loss
*γ*	No units	0.97	0	herbivorous protist assimilation efficiency
*c*	No units	1	3	capture probability
*m*_*z*,0_	day^-1^	0.05	0	herbivorous protist basal loss
*m*_*z*,*v*_	day^-1^	8.7 x 10^3^	1	herbivorous protist motility-based loss
*v*	μm day^-1^	1.73 x 10^6^	1	herbivorous protist swimming speed

**Table 3:** Variables based on a compilation of size-dependent relationships. Variables that are a function of several size-dependent relationships, as described in the main text.

Variable	Units	Description
*k*_*z*_	mM C	herbivorous protist grazing half saturation constant
*β*	μm^3^ s^-1^	encounter kernel
*D*_*p*_	μm^2^ s^-1^	phytoplankton diffusion coefficient
*D*_*z*_	μm^2^ s^-1^	herbivorous protist diffusion coefficient

### Phytoplankton variables and parameterization

Phytoplankton diameters varied from 1 to 512 μm on a logarithmic base 2 scale [37], creating ten size classes. Growth was modeled with a Holling II form [38]. Phytoplankton maximum growth rate μand nutrient uptake half saturation constant *k*_*s*_ (Table 2) had an allometric form [39] with values from [40]. Values from that study were nitrogen-based and so were converted to carbon for this study assuming Redfield ratio, 106 C:16 N (Table 2), to preserve consistency with other model parameters. Therefore, phytoplankton nutrient uptake of carbon *is constrained* by the amount of nitrogen that can be taken up. However, we acknowledge that carbon and nitrogen do not necessarily behave similarly, namely that nitrogen is generally limiting while carbon is not, and that these elements can be cycled through different processes (e.g., respiration). The general loss term *m*_*p*_ is a constant rate (Table 2) within the range of empirically measured loss values [41].

### Herbivorous protist variables and parameterization

Grazing explicitly represented several aspects of the feeding process, such as consumer motility and handling time, which can have significant impacts on predation rate [42–46]. Similar to phytoplankton, the distinguishing trait for consumers was cell diameter (Table 1), which we limited to three sizes 5, 50, and 200 μm. We chose these size classes specifically to be within the size range of protistan consumers [37]. To concisely highlight changes across this broad size range, we chose size classes that differ by an order of magnitude. We note that a finer size resolution is examined in the Supporting Information, which resulted in similar grazing dynamics as those described in the main text. Responses of intermediately sized consumers may lie somewhere between the results presented here; although, we do not assume linear dynamics.

Grazing (Eqs 1 and 2) was modeled with a Holling II form for which there is clear empirical support [47]. However, we acknowledge other functional forms could be employed [48, 49]. Grazing intake included the assimilation efficiency *γ*, parameterized based on empirical data as the average value at 15°C in [50] (Table 2).

In empirical [47, 51] and modeling [13, 21, 22] studies, maximum grazing rate typically decreases monotonically with grazer size and contains only implicit information about the prey. Given our aim to examine grazer preference for different prey sizes, we explicitly included both the prey and consumer sizes, *s*_*i*_ and *s*_*j*_, respectively:

$$\begin{array}{c} g\left(s_{i},s_{j}\right)=g_{0}(\frac{s_{j}}{s_{i}}{)}^{g_{e}}, \end{array}$$ (4)

The parameterization (Table 2) was based on empirical values synthesized in [40], revised to include the prey size S1 Fig, S1 Table.

Our model includes a specific representation of the handling time *h*. We modified the handling time formulation of [46] to allow consumers to handle prey larger than themselves [15–17]:

$$\begin{array}{c} h\left(s_{i},s_{j}\right)=h_{0}(\frac{s_{j}}{s_{i}}{)}^{h_{e}}, \end{array}$$ (5)

Handling time is often assumed to be the inverse of the maximum grazing rate *g*. However, an inverse of the empirically-based *g* values (S1 Fig, S1 Table) would mean handling time would *increase* as the consumer size increases relative to the prey size. Incongruously, the opposite pattern is a common formulation, namely that *h* decreases with consumer size and increases with prey size [46, 52–56]. Furthermore, indirect measurements like the maximum grazing rate can mask other grazing-related processes such as digestion [57]. Therefore, we examined the handling time parameterization with direct, empirical measurements for protistan consumers S2 Fig, S2 Table. While the relationship was not statistically significant (r^2^ = 0.1, p = 0.3), we used that parameterization here as it is likely due to measurement variability rather than a lack of an effect. We did perform a sensitivity analysis using the average handling time of 3.0x10^-3^ days S3 Fig. The general patterns still held; although, normalized biomass values were not always monotonically decreasing and grazing preferences were truncated for the 5-μm grazer and expanded for the multi-sized consumer system (Supporting information).

The grazing half saturation constant *k*_*z*_ was based on an analogy with enzyme kinetics [58]. The parameter was the ratio of the carbon content per prey cell *Q*_*p*,*i*_ and the encounter kernel *β*, handling time *h*, and capture probability *c*:

$$\begin{array}{c} k_{z}=\frac{Q_{p,i}}{\beta hc}, \end{array}$$ (6)

*Q*_*p*,*i*_ is the amount of carbon in the phytoplankton cell of size class i [59]. *β* is the encounter kernel, the volume of water in which the consumer can graze its prey [12]. The handling time *h* indicates the amount of time it takes for the consumer to process its prey [23], and *c* is the capture probability, the probability of a prey being encountered and not captured [46]. Below, we detail the formulation of each of these functions.

The carbon content per cell was modeled for a generic plankton [59] (Table 2). The capture probability [46] accounted for situations in which prey was encountered but not captured:

$$\begin{array}{c} c=(1-\frac{s_{i}}{s_{i}+s_{j}}{)}^{c_{e}}, \end{array}$$ (7)

This form was modified from [46] to allow diversity in predator prey sizes (Table 2). For a grazer of a certain size, the probability of capturing prey decreased with increasing prey size [53].

We assumed the encounter kernel (Table 3) depends on the processes of swimming and diffusion [12]. The encounter kernel for swimming was modeled as

$$\begin{array}{c} \beta_{v}=\pi (s_{i}+s_{j}{)}^{2}v, \end{array}$$ (8)

[12] for *v* the swimming speed of the grazer, which increased linearly with size, swimming 20 bodylengths s^-1^ (Table 2). For simplicity, prey did not swim. The diffusion encounter kernel took the form

$$\begin{array}{c} \beta_{D}=4\pi (D_{P}+D_{Z})(s_{i}+s_{j}), \end{array}$$ (9)

for *D*_*P*_ and *D*_*Z*_ the diffusion coefficients for phytoplankton and herbivorous protists, respectively (Table 3) [12]. The diffusion coefficient for both consumer and prey were calculated from $D=\frac{\kappa T}{3\pi \eta s}$ [12] for *κ* Boltzman’s constant, *T* temperature, *η* dynamic viscosity (see Supporting information). The final encounter kernel *β* [12] is:

$$\begin{array}{c} \beta =\beta_{v}+\beta_{D}, \end{array}$$ (10)

For a motile predator, *β*_*D*_ is negligible compared to *β*_*v*_.

Respiration was the sum of both a basal *R*_0_ and motility-associated *R*_*v*_ rate:

$$\begin{array}{c} R=R_{0}+R_{v}, \end{array}$$ (11)

*R*_0_ varied linearly with grazing intake, with a coefficient of 0.63 [50]. *R*_*v*_ was based on the idealized movement of a sphere of radius *r* through water with viscosity *η* at a velocity *v* [60]. Converting to a specific rate, the motility-based respiration had the form

$$\begin{array}{c} R_{v}=R_{v,0}\frac{s_{j}v^{2}\eta }{Q_{z,j}q}, \end{array}$$ (12)

for *R*_*v*,0_ a respiration coefficient of 0.041/day [60] and *q* the efficiency of transforming chemical work into mechanical work, which was taken as 1% [61]. *η* was as above for the diffusion coefficient (Supporting information).

Herbivorous protists mortality *m*_*z*_ was composed of a constant basal value *m*_*z*,0_ and a motility-associated mortality denoted *m*_*z*,*v*_, that increased with motility (Table 2) [62], leading to an increase in this parameter with increasing size. The total mortality *m*_*z*_ was the sum of these two rates:

$$\begin{array}{c} m_{z}=m_{z,0}+m_{z,v}, \end{array}$$ (13)

Overall, this model explicitly formulated trade-offs such that, for a given prey size, a larger consumer had the advantage of a shorter handling time and higher encounter probability compared to smaller consumers. However, larger organisms also had, for a given prey size, a lower maximum grazing rate and higher mortality rate compared to smaller consumers. Therefore, these parameterizations conferred trade-offs for different size grazers that *a priori* make their fitness implications unclear.

### Configuration of simulations

Using this model framework, we ran four model simulations. In three model runs, only one size class of grazer was present: 5, 50, or 200 μm in diameter. A fourth simulation included all three size classes of consumers together. All simulations were initialized with ten phytoplankton sizes. While we appreciate the effect of total nutrient, *N*_*T*_, concentration on planktonic size spectra [13, 63–65], we examined only one nutrient concentration, within the range of another size-based NPZ model with protistan consumers [40], to focus on grazing dynamics. Each model was integrated in time for ∼ 30 years with a 100-second time step and reached a steady-state in ∼7 years or less. The optimal prey size, defined as the phytoplankton size that led to the greatest biomass intake independent of loss, was determined at each time step and used as the prey choice for that entire time step. This formulation increased tractability and also allowed for adaptive feeding on a relatively short time scale, particularly in comparison to many grazing studies, which are generally on the order of several hours to a day (e.g., [66, 67]). The single size grazer systems showed constant biomass values in steady state. In the multiple sized grazer systems, the consumers and phytoplankton showed regular oscillations with a periodicity of 24.5 days S4 Fig. We focus on the average biomass values from the last three years of the 30-year model run for each system.

Using the averaged data, we examined the biomass distribution for the phytoplankton and herbivorous protist size classes. For the phytoplankton, the biomass per size class was normalized by the width of the size class. For the consumers, because there are only one or three size classes, we divided biomass values by the grazer size to obtain normalized values.

## Results

**Fig 1:**
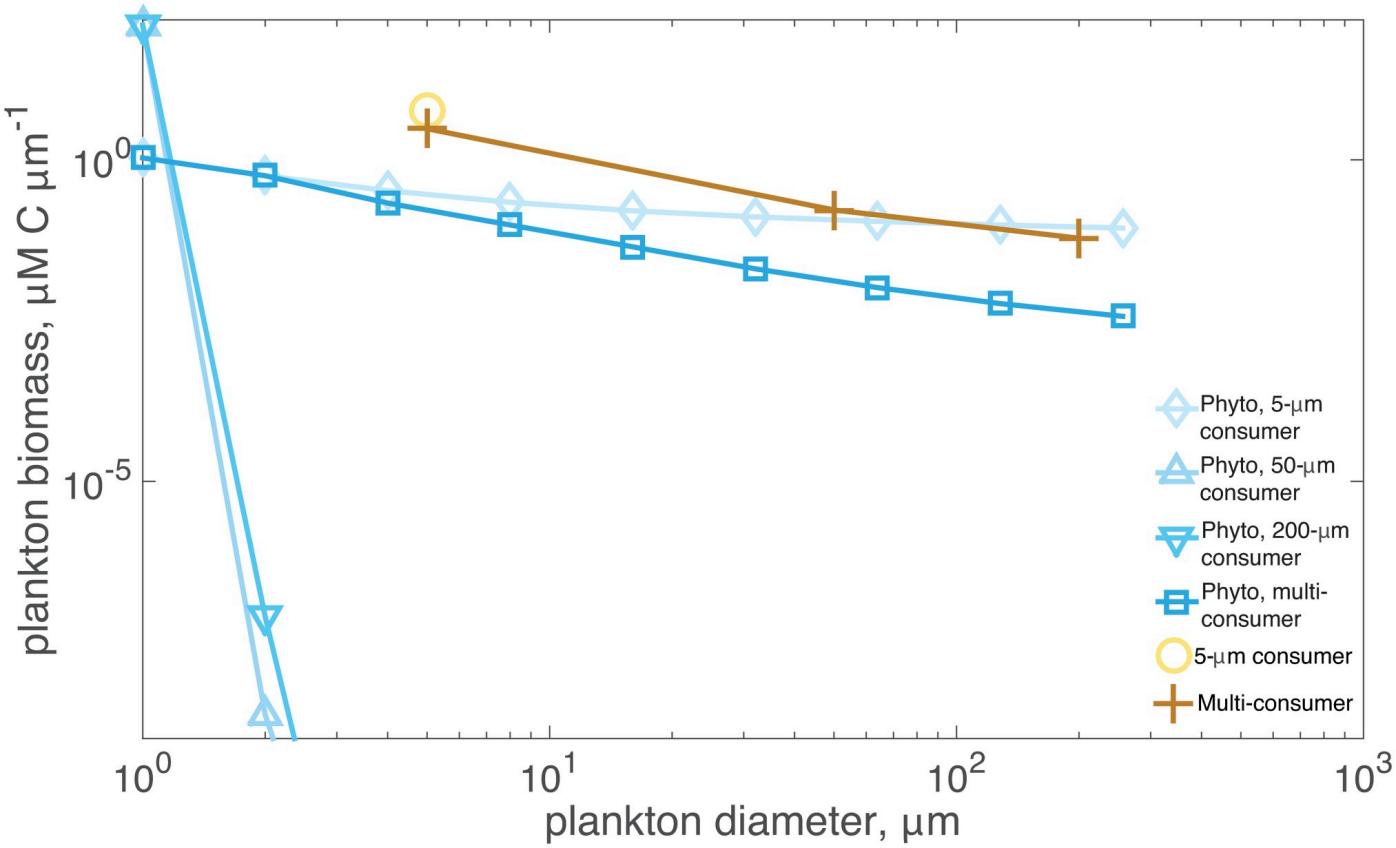
Planktonic biomass for nutrient-phytoplankton-zooplankton model systems with grazers of different sizes. The model systems include grazers of either 5, 50, or 200 μm diameter, and the multi-consumer model system corresponds to a system with all three sized consumers. The blue symbols correspond to the phytoplankton biomass for each of those systems (diamond for the 5-μm consumer system, upward triangle for 50-μm consumer, downward triangle for 200-μm consumer, and square for multi-consumer system). The yellow symbols correspond to consumer normalized biomass values (circle for the 5-μm consumer, and plus sign for the multi-consumer system). The 50- and 200-μm grazers did not survive in single consumer size class simulations and thus no results are shown.

Under predation pressure by only the smallest protists (5 μm) phytoplankton biomass was distributed throughout all size classes (Fig 1). Normalized phytoplankton biomass values ranged from 1.07 μM C μm^-1^ to 0.09 μM C μm^-1^, decreasing with increasing size (Fig 1). The normalized consumer biomass was 5.85 μM C μm^-1^ (Fig 1). The grazer-prey biomass ratio was 0.31.

**Fig 2:**
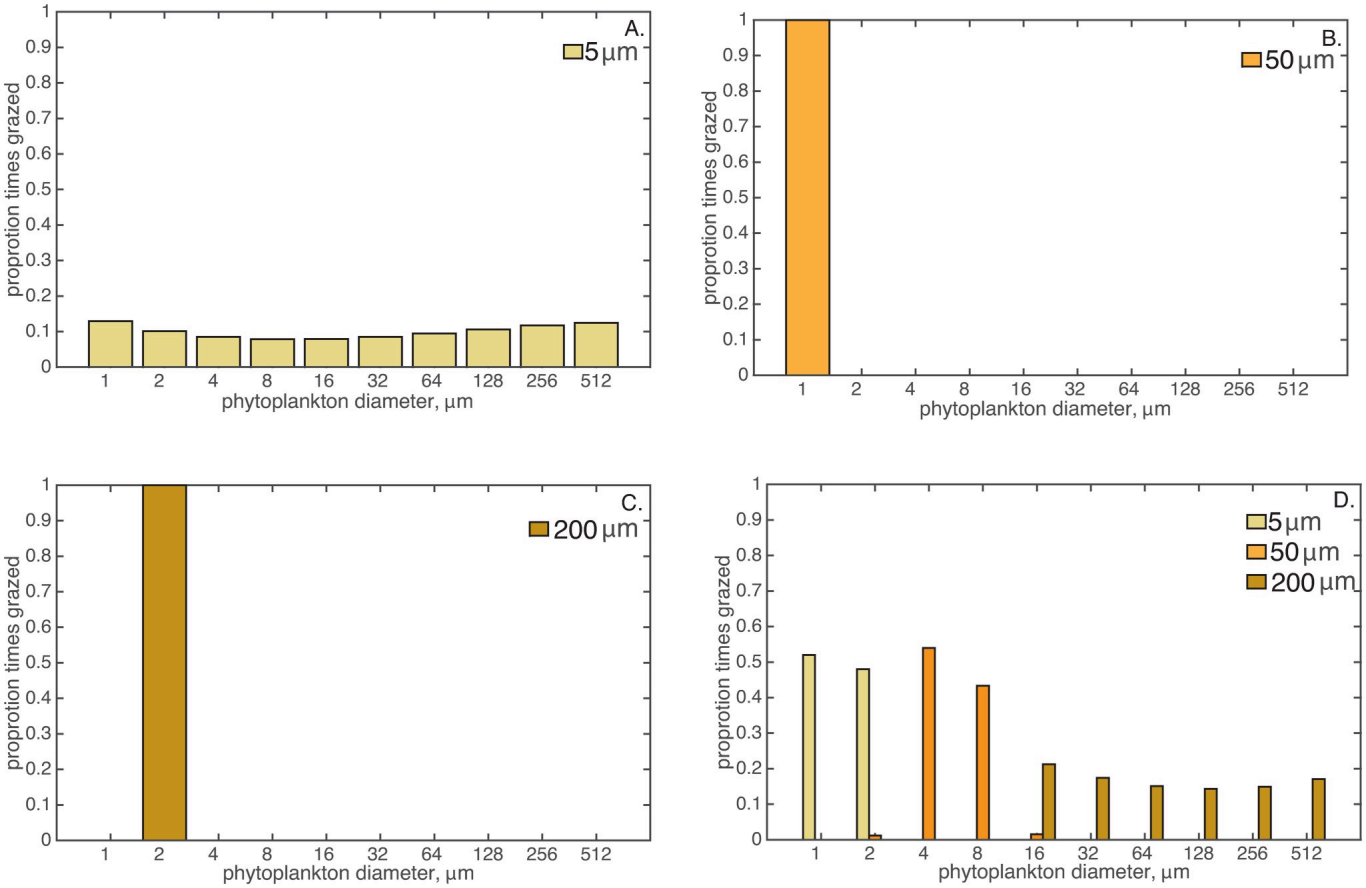
Proportion of times consumers grazed different sized phytoplankton during the last 3 years of a ∼ 30 year model run time. Model run in which the grazers are all a. 5-μm, b. 50-μm or c. 200-μm in diameter, and d. multi-size class grazers with 5-, 50-, and 200-μm grazers combined.

To investigate prey size selection, we calculated the proportion of times each prey size class was grazed in the last three years of the model run (Fig 2). The 5-μm grazers consumed all prey size classes, with generally similar preferences for all size classes, 13% of the time for the most (1-μm) and 8% for the least (8-μm) frequently grazed phytoplankton (Fig 2a). These grazing preferences correspond to an inversion of consumer-prey size ratios from 5:512 (∼ 0.01: 1) to 5:1. While this prey size range is extremely broad, these results highlight the underlying dynamics, namely generalist prey size selection, that impact the size diversity of the system when compared to the other model runs, described below.

For scenarios including the 50- or 200-μm grazers, they exclusively grazed the 1- and 2-μm phytoplankton size classes, respectively (Fig 2b and 2c), corresponding to 50:1 and 100:1 grazer-prey size ratios, respectively. However, the consumers were not able to persist (biomass < 10^−6^ μM μm^-1^) in this generalized, size-dependent model for protistan consumers since the smallest producers dominated production but were too small to meet the nutritional requirements of the large predators. Consequently, only the smallest 1-μm phytoplankton size class had appreciable biomass, 125 μM C μm^-1^, in the steady-state solution (Fig 1).

Diversity in grazer size classes imparted stability on the community composition: when all three grazer classes were present, they co-existed along with all phytoplankton size classes (Fig 1). The normalized phytoplankton size spectrum decreased monotonically from 1.1 μM C μm^-1^ to 3.7x10^-3^ μM C μm^-1^. The normalized grazer biomass values were 3.05, 0.17, and 0.06 μM C μm^-1^ for the 5-, 50-, and 200-μm consumers, respectively (Fig 1). The grazer-prey biomass ratio was 4.1.

Consumer grazing preferences in the multi-consumer simulation were different than the single-sized grazer systems (Fig 2d). The 5-μm consumer preferred the two smallest phytoplankton size classes, 1 and 2 μm, in approximately equal amounts of 52% and 48%, respectively. The 50-μm consumer preferentially consumed the 4- and 8-μm phytoplankton in roughly equal amounts, about 54% and 43% of the time, respectively. The 50-μm consumer occasionally consumed the 2- and 16-μm size classes, each about 1% of the time. The largest 200-μm consumer preferred the 16-μm size class of phytoplankton, grazing it about 21% of the time. The largest grazer also consumed all larger phytoplankton, between 17% and 14% of the time. The grazer-prey size ratios varied from 25:1 to 200:512 (∼ 0.4: 1).

**Fig 3:**
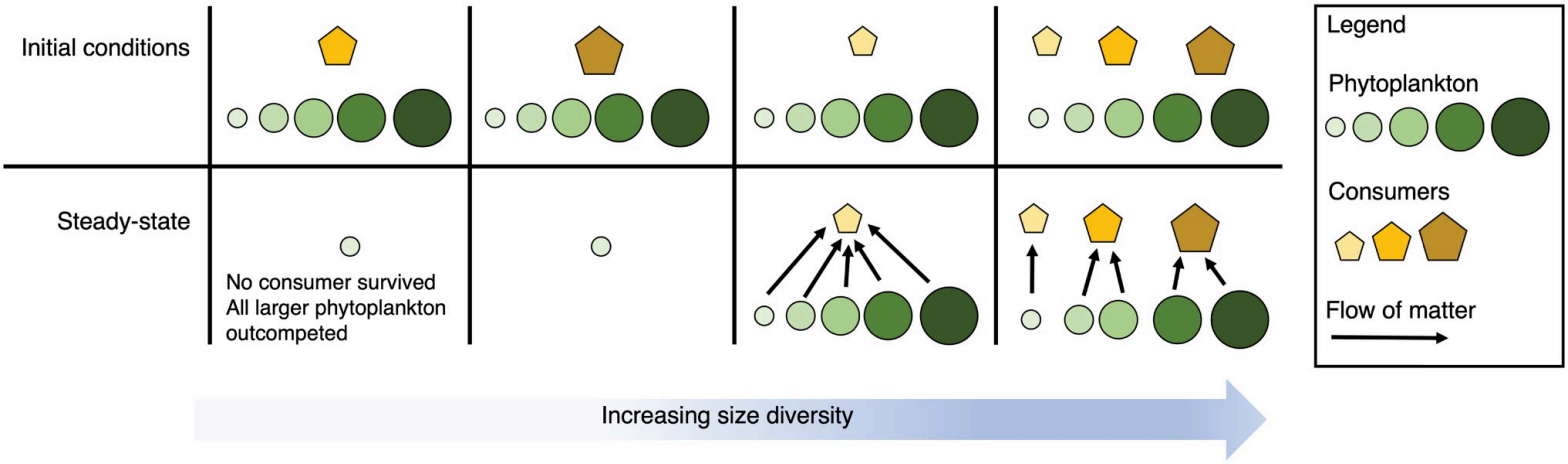
Changes in the size diversity of the planktonic community among four modeled systems, with each of three consumers separately or all together. Initial conditions are in the top panels and steady-state solutions in the bottom panels. A keystone predator effect facilitates competitive abilities of different size classed phytoplankton. The most diverse grazer community supports a diverse phytoplankton community. Sizes of symbols are relative sizes and not to scale.

Comparing among these modeled systems, the allometric scaling of phytoplankton parameters allowed the smallest phytoplankton to outcompete larger phytoplankton when relieved of top-down control (Fig 3). When consumers did persist, they were able, either singly or collectively, to graze all size classes and increase the size diversity of their systems. This pattern persisted even with a constant handling time and when eight consumer size classes were considered (Supporting information).

## Discussion

Using an empirically motivated framework that models the mechanisms underlying feeding, we examined the emergent plankton abundance and size structure as a function of grazing behavior of herbivorous protists. This approach is in the same vein as the diet breadth [68] and allometric diet breadth models [69, 70]. In our study, we modeled, separately and together, three different sized consumers that each had the ability to graze any phytoplankton prey. This flexible framework, as opposed to a more rigid, prescribed formulation of grazing, allowed us to examine the interplay between emergent prey size choice and phytoplankton community structure. Comparing the different systems, some fundamental features became apparent, such as distinct grazer-prey size ratios, the keystone predator effect, and dietary niche partitioning.

The canonical 10:1 predator-prey size ratio [11, 12] did not systematically emerge, but the simulated range when all grazer sizes co-existed (25:1 to 0.4:1) encompassed this often-assumed value. Inversions with prey size exceeding consumer size were also supported, as has been observed among protistan consumers [15–17]. Prey size selection was dynamic and depended on grazer size and whether other grazers were present. Prey size selection, in turn, had strong implications for consumer and prey survival and, consequently, the diversity of the system.

When grazers did not consume enough prey to survive in this protistan model formulation, only the smallest, most competitive phytoplankton persisted [65]. When consumers did survive, they grazed the smallest, most competitive prey, freeing up resources for the larger, less competitive phytoplankton [71]. Larger phytoplankton grew until they were abundant enough to be grazed. Thus, the balance between top-down and bottom-up forcing at equilibrium determined the prey size distribution [22].

This top-down control resembled the keystone predator effect [31, 32]. In the most general sense, a keystone predator allows competing prey to coexist [72]. More specifically, a keystone predator consumes the most competitive prey, resulting in the survival of less competitive prey. The concept was introduced in marine intertidal and aquatic settings [31, 73] and has since been observed in aquatic and terrestrial ecosystems [72, 74, 75] and recreated in modeling studies [32, 76]. Similar to those studies, the model system in this study indicated that selective feeding by consumers increased the size-diversity of prey by grazing the smallest, most competitive prey, subsequently releasing resources for larger phytoplankton. Thus, more size classes coexisted when all size classes were grazed.

Another ecological property that emerged specifically from the multiple-size grazer system was dietary niche partitioning [33, 34]. That is, each size consumer selected largely non-overlapping sizes of phytoplankton to graze. The emergent, distinct grazing size preferences persisted even when eight sized grazers were included S5 Fig.

Our modeled results were similar to the empirical observations of the impact of zooplankton on phytoplankton in the East China Sea [77]. In that study, the size diversity of mesozooplankton was the most important factor determining the top-down control on phytoplankton, which was attributed to dietary niche partitioning.

When examining protistan grazers, there is strong evidence for size selective grazing [14, 78–80], which can lead to resource niche partitioning [55]. However, the impact of selective feeding on the planktonic community structure can be more varied [55, 66, 81]. For example, in a microcosm experiment, two protistan grazers showed coexistence by size-selective resource partitioning [55]. While grazer size-selection changed the prey community structure, there was no consequent change in biomass. Our multiple sized grazer system also showed emergent size-selective feeding, but we saw an increase in total grazer biomass compared to the single grazer systems. Therefore, our model results are more in alignment with the idea of complementarity [82] in which ecosystem function increases with increased diversity.

In all our modeled systems, the normalized phytoplankton biomass spectrum decreased as the size of the phytoplankton increased, similar to natural ecosystems [28, 29]. When the normalized phytoplankton biomass spectrum from the multi-sized consumer system was converted to units of cells per ml per size class, the spectral slope was -3.97. This slope is remarkably similar to the spectral slope of -4 for field data from stable mesotrophic and oligotrophic environments ([30] and references therein). This size distribution is noticeably steeper than that proposed by the metabolic theory of ecology for a trophic level [83].

The shape of the phytoplankton biomass spectra was attributed to consumers that grazed larger sized phytoplankton additionally grazing larger size *ranges* of phytoplankton [30]. This patterning, supported by empirical measurements [36], was an emergent property of the modeled multi-sized consumer system in this study and held even when more size classes of consumers were included S5 Fig. However, the steady-state biomass for the phytoplankton in this study’s modeled systems was not only due to grazing pressure but also to phytoplankton competitive ability, which was made clear from a comparison with the single-grazer systems, thus broadening the factors that need to be considered and experimentally investigated.

Overall, this study’s model formulation includes flexible grazing behavior based on first principles, which allowed us to investigate the impact of top-down processes in structuring the community without the level of prescription common in planktonic grazer models. The framework shown here could be used to accommodate more complex interactions and processes, such as different resource acquisition strategies and feeding modes [54, 84] and changes in nutrient content of prey [24, 85] or trade-offs between phytoplankton competitive ability and grazer defenses, including mixotrophic species [86]. These detailed representations of complex grazer dynamics have highlighted important, grazer mediated forcing functions that structure phytoplankton communities, which have ramifications for biogeochemical cycles.

## Supporting information

S1 Fig Regression of the ratio of consumer and prey radius against maximum grazing rate. (TIF)

S2 Fig Regression of the ratio of consumer and prey radius against handling time. (TIF)

S3 Fig Normalized phytoplankton and grazer biomass and size classes grazed for four different systems in which the grazers are 5-, 50- or 200-μm in diameter, or all three grazers sizes are present. A. Normalized biomass. The blue symbols represent normalized phytoplankton biomass, and the yellow symbols correspond to consumer normalized biomass. No consumers survived when they were only of 50 or 200 μm in size, and thus those biomass values are not shown. B-E. Proportion of times each size class was grazed by the 5-μm consumer (B), 50-μm consumer (C), 200-μm consumer (D), and all consumers together (E).
(TIF)

S4 Fig Time-dependent normalized phytoplankton and protistan grazer biomass values for last three years of model run. A. 5 μm grazer system. B. 50- and 200-μm grazer systems. Note that only the 1-μm phytoplankton survived in both systems, and only those two groups are shown. C. Multi-sized consumer system.
(TIF)

S5 Fig Community structure for a modeled system with grazers of eight different sizes. A. Normalized plankton size spectra. B. prey size selections for each consumer.
(TIF)

S1 Table Measurements of maximum grazing rate. (PDF)

S2 Table Handling time measurements. Values for handling time, consumer radius, and prey radius were taken from the same source. The only exception is that the prey radius for *Ochromonas* in (Fenchel 1982a) which came from (Fenchel 1982b).
(PDF)
