# Supplementary figures and images for: Planktonic prey size selection reveals an emergent keystone predator effect and niche partitioning
Source: PLoS One. 2023 Feb 13;18(2):e0280884. doi: 10.1371/journal.pone.0280884 (PMC9925011; doi:10.1371/journal.pone.0280884)

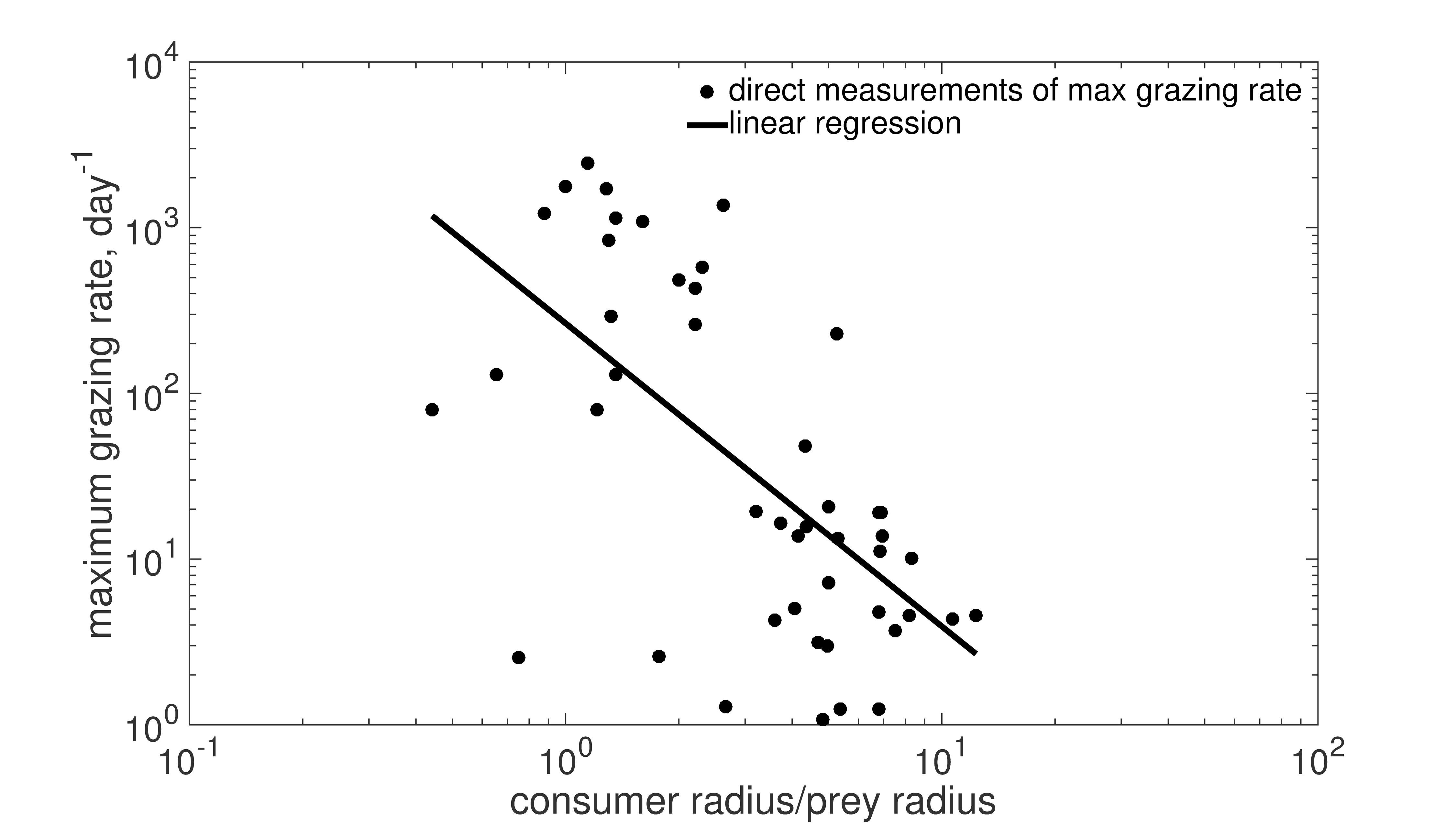

Supplement: S1 Fig — (TIF) [file pone.0280884.s001.tif]

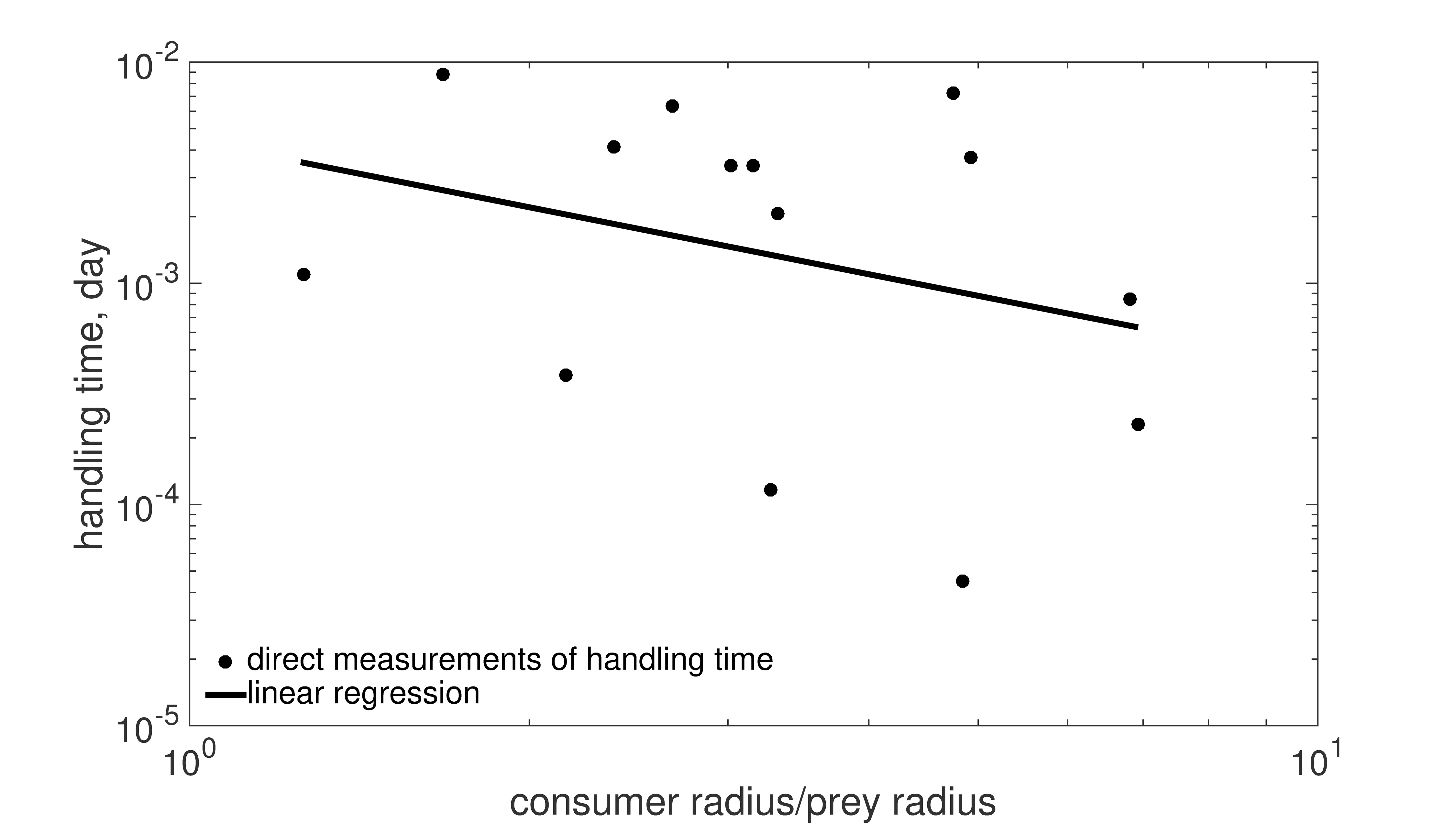

Supplement: S2 Fig — (TIF) [file pone.0280884.s002.tif]

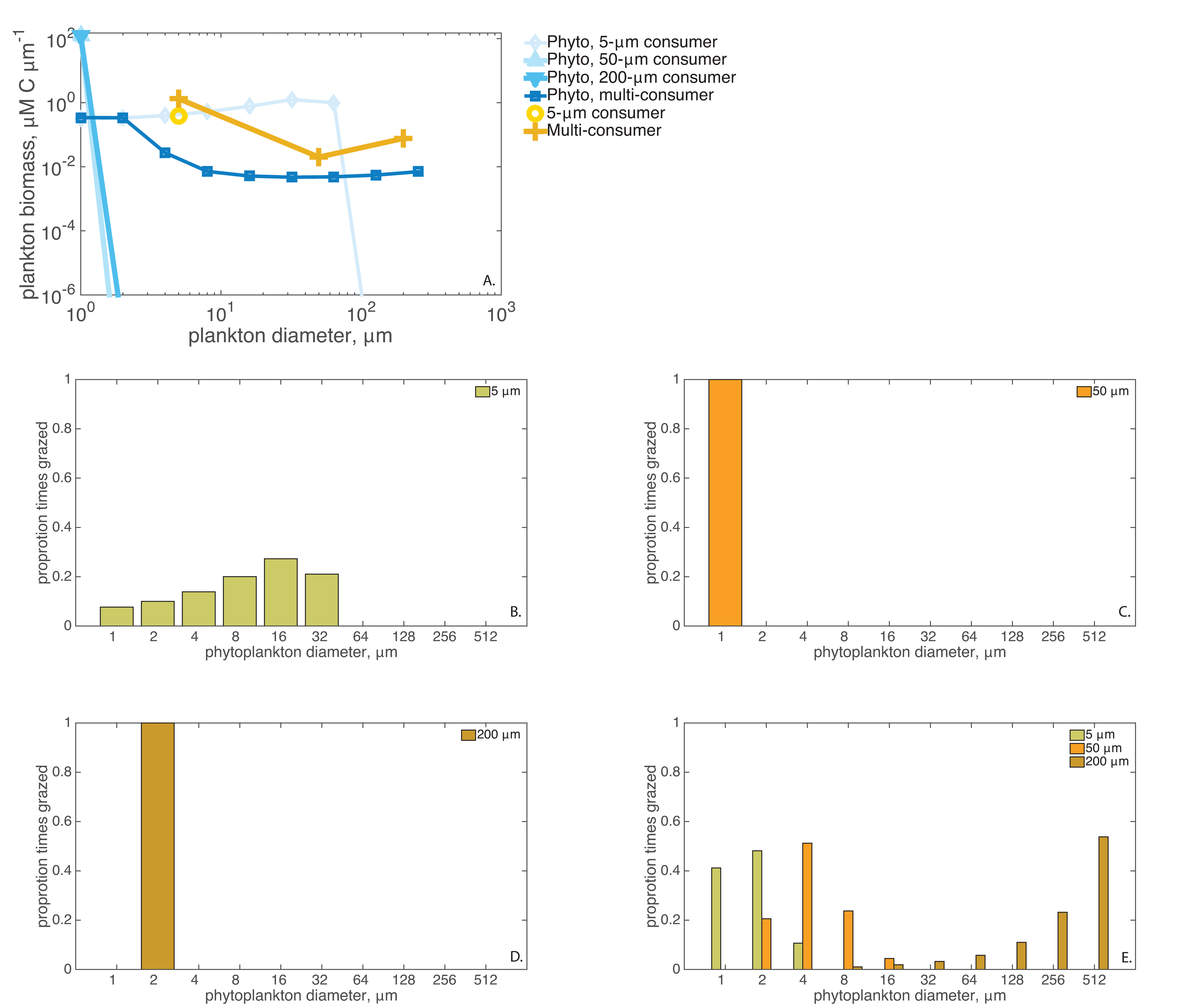

Supplement: S3 Fig — A. Normalized biomass. The blue symbols represent normalized phytoplankton biomass, and the yellow symbols correspond to consumer normalized biomass. No consumers survived when they were only of 50 or 200 μm in size, and thus those biomass values are not shown. B-E. Proportion of times each size class was grazed by the 5-μm consumer (B), 50-μm consumer (C), 200-μm consumer (D), and all consumers together (E). (TIF) [file pone.0280884.s003.tif]

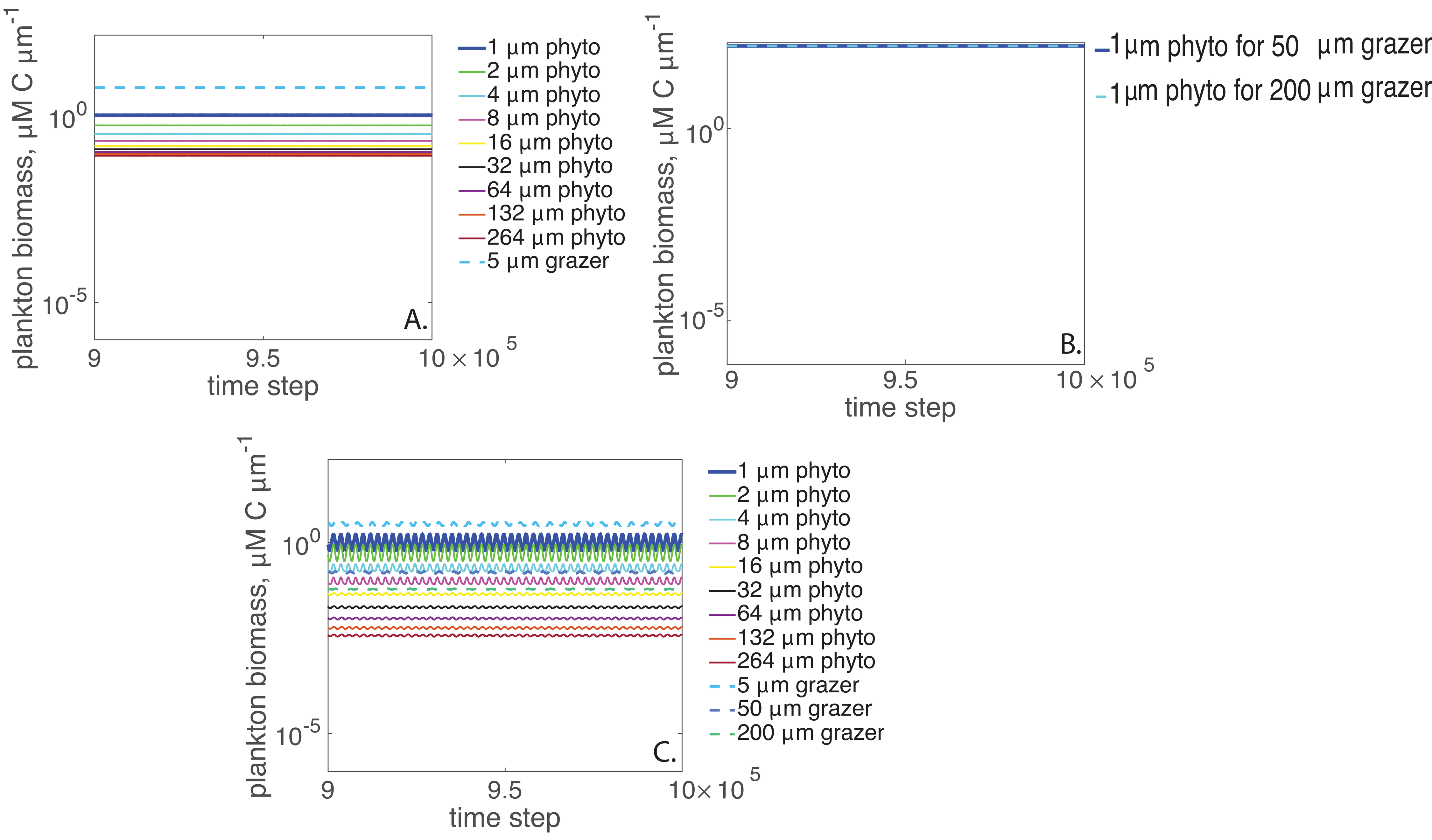

Supplement: S4 Fig — A. 5 μm grazer system. B. 50- and 200-μm grazer systems. Note that only the 1-μm phytoplankton survived in both systems, and only those two groups are shown. C. Multi-sized consumer system. (TIF) [file pone.0280884.s004.tif]

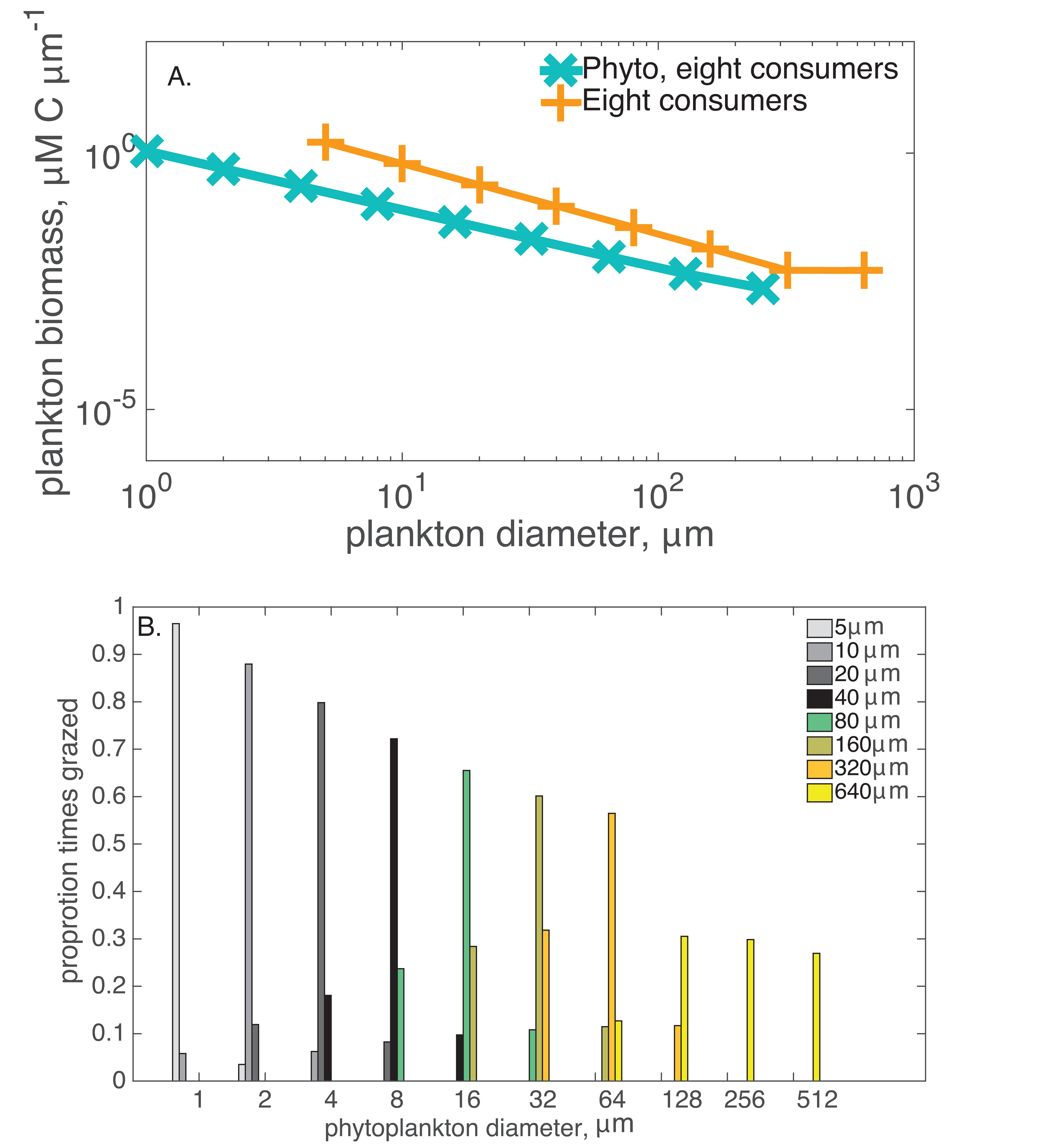

Supplement: S5 Fig — A. Normalized plankton size spectra. B. prey size selections for each consumer. (TIF) [file pone.0280884.s005.tif]
