# Supplementary material for: Planktonic prey size selection reveals an emergent keystone predator effect and niche partitioning
Source: PLoS One. 2023 Feb 13;18(2):e0280884. doi: 10.1371/journal.pone.0280884 (PMC9925011; doi:10.1371/journal.pone.0280884)
Supplement: S1 Table — (PDF) [file pone.0280884.s006.pdf]

Consumer	Consumer radius (μm)	Prey	Prey radius (μm)	Max grazing rate, 1/day	Source for max grazing rate	Source for consumer size	Source for prey size
Gymnodinium sp.	6.00	*Rhodomonas salina*	8.00	0.04	[4]	[4]	[4]
Protoperidinium bipes	7.80	*Skeletonema costatum*	5.90	2.90	[4]	[4]	[4]
Protoperidinium hirobis	8.70	*Leptocylindrum danicus*	19.70	0.80	[4]	[4]	[4]
Luciella masanensis	13.50	perch bloods	6.10	2.60	[4]	[4]	[4]
Pfiesteria piscicida	13.50	perch bloods	6.10	4.30	[4]	[4]	[4]
Stoeckeria algicida	13.90	*Heterosigma akashiwo*	11.50	0.80	[4]	[4]	[4]
Oxyrrhis marina	15.60	*Heterosigma akashiwo*	11.50	1.30	[4]	[4]	[4]
Protoperidinium vorax	21.00	*Skeletonema pseudocostatum*	4.00	2.30	[4]	[4]	[4]
Oblea rutunda	21.60	*Ditylum brightwellii*	33.00	1.30	[4]	[4]	[4]
Protoperidinium huberi	26.50	*Ditylum brightwellii*	26.50	17.80	[4]	[4]	[4]
Diplopsalis lenticula	31.00	*Ditylum brightwellii*	35.20	19.40	[4]	[4]	[4]
Gyrodinium spirale	31.80	*Prorocentrum minimum*	12.10	13.60	[4]	[4]	[4]
Protoperidinium pellucidum	36.10	*Ditylum brightwellii*	26.50	11.50	[4]	[4]	[4]
Polykrikos kofoidii	43.50	*Gymnodinium catenatum*	34.00	17.10	[4]	[4]	[4]
Polykrikos kofoidii	43.50	*Lingulodinium polyedrum*	37.90	24.40	[4]	[4]	[4]
Protoperidinium conicum	45.70	*Ditylum brightwellii*	35.20	17.70	[4]	[4]	[4]
Protoperidinium divergens	61.00	*Lingulodinium polyedrum*	38.20	12.00	[4]	[4]	[4]
Protoperidinium crassipes	73.00	*Lingulodinium polyedrum*	36.60	5.30	[4]	[4]	[4]
Protoperidinium depressum	81.00	*Ditylum brightwellii*	35.20	12.10	[4]	[4]	[4]
Tintinnopsis dadayi	59.98	*Katodinium rotundatum*	8.00	3.36	[5]	[6]	[5]
Strobilidium cf. spiralis	36.99	*Pseudobodo cf. trumulans*	4.50	3.36	[5]	[6]	[5]
Actinomonas mirabilis	2.62	*Pseudomonas spp.*	0.52	20.64	[6]	[6]	[7]
Monosiga sp.	1.68	*Pseudomonas spp.*	0.52	19.44	[6]	[6]	[7]
Ochromonas sp.	3.63	*Pseudomonas spp.*	0.52	13.68	[6]	[6]	[7]
Paraphysomas vestita	3.57	*Pseudomonas spp.*	0.52	19.20	[6]	[6]	[7]
Pleoromonas jaculans	2.29	*Pseudomonas spp.*	0.52	15.60	[6]	[6]	[7]

Pseudobodo tremulans	2.78	*Pseudomonas spp.*	0.52	13.44	[6]	[6]	[7]
Bodo designis	2.34	*Alteromonas or Shewanella*	0.54	47.76	[6]	[6]	[8]
Ciliophrys infusionum	3.74	*Alteromonas or Shewanella*	0.54	18.96	[6]	[6]	[8]
Codosiga gracilis	2.03	*Alteromonas or Shewanella*	0.54	16.56	[6]	[6]	[8]
Jakoba libera	2.62	*Alteromonas or Shewanella*	0.54	1.08	[6]	[6]	[8]
Paraphysomonas iperforata	3.70	*Alteromonas or Shewanella*	0.54	4.80	[6]	[6]	[8]
Stephanoeca diplocostata	2.71	*Alteromonas or Shewanella*	0.54	7.20	[6]	[6]	[8]
Diaphanoeca grandis	2.12	*Pseudomonas sp.*	0.52	7.92	[6]	[6]	[9]
Ochromonas sp.	2.29	*Escherichia coli*	0.55	10.08	[6]	[6]	[10]
Spumella sp.	2.50	*Rod-shaped bacteria*	0.50	1.92	[6]	[6]	[11]
Gymnodinium sp.	5.99	*Isochrysis galbana*	2.25	2.69	[6]	[6]	[12]
Gyrodinium spirale	14.00	*Heterocapsa triquetra*	7.88	4.08	[6]	[6]	[13]
Favella ehrenbergii	36.87	*Heterocapsa triquetra*	7.88	3.12	[6]	[6]	[13]
Favella ehrenbergii	28.37	*Heterocapsa triquetra*	7.88	5.16	[6]	[6]	[14]
Lohmaniella spiralis	32.96	Latex beads	4.85	2.64	[6]	[6]	[14]
Strombidium reticulatum	21.22	*Tetraselmis suecica*	3.95	2.64	[6]	[6]	[14]
Strombiilidium cf. spiralis	18.47	*Isochrysis galbana*	1.50	4.54	[6]	[6]	[5]
Strombiilidium cf. spiralis	18.47	*Pseudobodo*	2.25	4.54	[6]	[6]	[5]
Tintinnopsis dadayi	29.99	*Katodinium rotundatum*	4.00	3.72	[6]	[6]	[5]
Strombidium sulcatum	13.37	*Dunaliella minuta*	3.22	11.54	[6]	[6]	[15]
Strombidium sulcatum	13.37	*Monochrysis lutheri*	1.95	9.38	[6]	[6]	[15]
S*trombidium sulcatum*	13.37	*Nannochloris* sp.	1.25	3.62	[6]	[6]	[15]
