# Supplementary material for: Planktonic prey size selection reveals an emergent keystone predator effect and niche partitioning
Source: PLoS One. 2023 Feb 13;18(2):e0280884. doi: 10.1371/journal.pone.0280884 (PMC9925011; doi:10.1371/journal.pone.0280884)
Supplement: S2 Table — Values for handling time, consumer radius, and prey radius were taken from the same source. The only exception is that the prey radius for Ochromonas in (Fenchel 1982a) which came from (Fenchel 1982b). (PDF) [file pone.0280884.s007.pdf]

Consumer	Consumer radius (μm)	Prey	Prey radius (μm)	Handling time, day	Source for handling time	Source for consumer size	Source for prey size
Cafeteria roenbergensis	1.6407	*Pseudomonas putida*	1.3	0.00109375	[16]	[16]	[16]
Bodo saltans	1.8331	*Pseudomonas putida*	0.85	0.00038426	[16]	[16]	[16]
Spumella sp.	2.6208	*Pseudomonas putida*	0.8	0.0001169	[16]	[16]	[16]
Ochromonas sp.	3.3887	*Pseudomonas putida*	0.7	4.4792E-05	[16]	[16]	[16]
Ochromonas	3.625	*Pseudomonas*	0.5232	0.00023148	[17]	[17]	[7]
Prorocentrum donghaiense	6.65	*Cryptophyte*	2.8	0.00413194	[18]	[18]	[18]
Heterocapsa triquetra	7.5	*Cryptophyte*	2.8	0.00635417	[18]	[18]	[18]
Prorocentrum micans	13.3	*Cryptophyte*	2.8	0.00728009	[18]	[18]	[18]
Lingulodinium polyedrum	19.1	*Cryptophyte*	2.8	0.00084491	[18]	[18]	[18]
Lingulodinium polyedrum	19.1	*Heterosigma akashiwo*	5.75	0.00206019	[18]	[18]	[18]
Lingulodinium polyedrum	19.1	*Prorocentrum minimum*	6.05	0.0033912	[18]	[18]	[18]
Lingulodinium polyedrum	19.1	*Scrippsiella trochoidea*	11.4	0.00875	[18]	[18]	[18]
Gonyaulax polygramma	16.25	*Amphidinium carterae*	3.3	0.00368634	[19]	[19]	[19]
Gymnodinium aureolum	8.4452	*Teleaulax sp.*	2.8	0.00344907	[20]	[20]	[20]
